# New phenotyping questionnaire for diagnosing sarcoidosis-associated small fiber neuropathy

**DOI:** 10.1093/braincomms/fcae289

**Published:** 2024-08-28

**Authors:** Lisette R M Raasing, Oscar J M Vogels, Mirjam Datema, Carmen A Ambarus, Martijn R Tannemaat, Jan C Grutters, Marcel Veltkamp

**Affiliations:** ILD Center of Excellence, Department of Pulmonology, St Antonius Hospital, 3435 CM Nieuwegein, The Netherlands; ILD Center of Excellence, Department of Neurology, St Antonius Hospital, 3435 CM Nieuwegein, The Netherlands; ILD Center of Excellence, Department of Clinical Neurophysiology, St Antonius Hospital, 3435 CM Nieuwegein, The Netherlands; ILD Center of Excellence, Department of Pathology, St Antonius Hospital, 3435 CM Nieuwegein, The Netherlands; Department of Clinical Neurophysiology, Leiden University Medical Center, 2333 ZA Leiden, The Netherlands; ILD Center of Excellence, Department of Pulmonology, St Antonius Hospital, 3435 CM Nieuwegein, The Netherlands; Division of Heart and Lungs, University Medical Center Utrecht, 3584 CX Utrecht, The Netherlands; ILD Center of Excellence, Department of Pulmonology, St Antonius Hospital, 3435 CM Nieuwegein, The Netherlands; Division of Heart and Lungs, University Medical Center Utrecht, 3584 CX Utrecht, The Netherlands

**Keywords:** thermal threshold testing, sensory testing, nerve fiber density, SFNSL, pain

## Abstract

Small fiber neuropathy is a common complication in patients with sarcoidosis and its prevalence is estimated at 40–86%. The underlying mechanism influences the presentation of small fiber neuropathy. For example, patients with metabolic diseases are often associated with a classic length-dependent small fiber neuropathy pattern, while patients with inflammatory diseases are more often present with a non-length-dependent small fiber neuropathy. Detailed phenotyping may be useful to improve diagnostic efficiency, as a clue to underlying mechanisms and as a precondition for personalized medicine. This study examined four phenotypes distinguishing between length-dependent and non-length-dependent presentation with a new subdivision for continuous and intermittent presentation. Forty-eight sarcoid patients with symptoms and at least two clinical signs of small fiber neuropathy and normal nerve conduction studies were classified as having probable small fiber neuropathy. A new small fiber neuropathy phenotyping questionnaire has been developed that allows patients to mark the anatomical locations of pain at three different levels: the skin, muscles, and joints. The location of symptoms was used to define length dependence, and two colors were used to distinguish continuous (red) from intermittent (blue) symptoms. In addition, skin biopsy, corneal confocal microscopy, Sudoscan and water immersion skin wrinkling were used to investigate a correlation between the four phenotypes, sensory function, nerve fiber density, and autonomic nerve function. Overall, 35% of patients with probable small fiber neuropathy showed length-dependent symptoms and 44% showed non-length-dependent symptoms while 21% suffered from non-neuropathic musculoskeletal pain. The distinction between intermittent and continuous symptoms showed significantly less continuous than intermittent non-length-dependent symptoms (odds ratio = 0.3, *P* = 0.01). Moreover, continuous length-dependent symptoms were the only phenotype that correlated with thermal threshold testing (*R* = 0.3; *P* = 0.02) and the small fiber neuropathy screening list (*R* = 0.3; *P* = 0.03). In addition, thermal threshold testing (TTT) also correlated with the small fiber neuropathy (SFN) screening list (*R* = 0.3; *P* = 0.03). Other diagnostic methods showed no correlation with any of the four defined phenotypes. A novel finding is that TTT is only associated with continuous length-dependent pain, suggesting that TTT could result in more false negatives in patients with other pain phenotypes. Determining the pathophysiologic mechanisms could help develop new diagnostic methods. If patients suspected of SFN show symptoms without a length-dependent continuous presentation, the diagnosis should focus less on the diagnostic methods used.

## Teaser

Raasing *et al*. investigated novel phenotypes based on patient-reported pain in patients with sarcoidosis-associated small fiber neuropathy. They distinguished between continuous, intermittent, length-dependent, and non-length-dependent phenotypes. Patient-reported continuous non-length-dependent pain was less prevalent, and continuous length-dependent pain was the only phenotype associated with thermal threshold testing.

## Graphical Abstract

**Graphical abstract:**
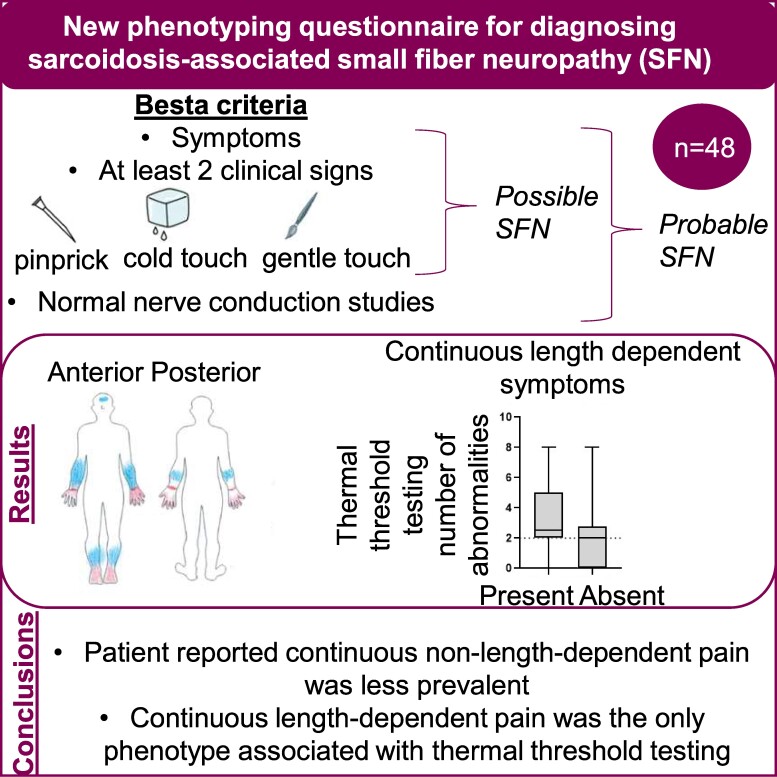

## Introduction

Small fiber neuropathy (SFN) is a heterogeneous disorder affecting the Aδ and C-fibers.^1^ SFN affects small sensory and autonomic fibers, resulting in neuropathic pain, burning sensations, allodynia, bedsheet intolerance, sweating abnormalities, gastrointestinal dysmotility, or orthostasic hypotension.^2-7^ The pathophysiology of SFN is poorly understood. It is associated with a variety of diseases, including diabetes, infections, inflammatory disorders, and genetic abnormalities such as mutations in the sodium channels Na(V)1.7–1.9.^1,8^ The underlying disease influences clinical manifestations of SFN in two different ways. First, patients with metabolic disease often present with classical length-dependent SFN,^9^ while patients with immune-mediated diseases more often present with non-length-dependent SFN.^10^ Second, patients with sodium channel mutations typically present with intermittent rather than continuous symptoms, similar to a paroxysmal extreme pain disorder.^8^

Based on the heterogeneous clinical manifestations, diagnosing SFN remains a major challenge. In addition to genetic testing, several diagnostic tests have been developed to assess small nerve fibers, by determining nerve fiber density, sensory function, or autonomic function.^11^ A gold standard is currently lacking, but diagnostic criteria including intraepidermal nerve fiber density (IENFD) and thermal threshold testing (TTT) are widely recognized as useful modalities.^12^ Because both IENFD and TTT are measured distally, there is a risk of underdiagnosis of SFN in patients with non-length-dependent SFN.^13^ Corneal confocal microscopy (CCM), which measures corneal nerve fiber density (CNFD), has been suggested as a supplementary tool to specifically assess non-length-dependent nerve fiber loss.^14^ In addition, autonomic function tests show added value in diagnosing SFN.^15^

Sarcoidosis is an immune-mediated granulomatous disorder of unknown cause, mainly affecting the lungs and lymph nodes.^16^ SFN is a common complication in patients with sarcoidosis estimated to occur in 40–86%.^17,18^ The exact prevalence of length-dependent and non-length-dependent pain in sarcoidosis-associated SFN (SSFN) is unknown. Data on intermittent or continuous presentation of SFN-related symptoms are also lacking as this is not specifically addressed in the SFN screening list (SFNSL).^19^ To improve the diagnosis of SSFN, a better understanding of patient-reported symptoms is important, which prompted us to conduct current research.

The purpose of this study is threefold. First, we aimed to determine the prevalence of length-dependent and non-length-dependent patient-reported pain in patients with SSFN. Second, we examined the prevalence of patient-reported intermittent and continuous pain. Finally, we tested whether different diagnostic modalities were associated with specific patient-reported pain presentations (length-dependent, non-length-dependent, continuous, or intermittent).

## Materials and methods

### Ethics

The local Ethics Committee (Medical Research Ethics Committees United, Nieuwegein, the Netherlands R19.080) approved our study. Verbal and written consent was obtained before the start of the study. Furthermore, the study was conducted according to the Declaration of Helsinki and GCP guidelines.

### Design

This was a prospective, cross-sectional, and observational study, conducted from January 2021 to September 2022 at the outpatient clinic of St. Antonius Hospital, a tertiary referral center for sarcoidosis and interstitial lung diseases (ILD) in the Netherlands. Patients with sarcoidosis with symptoms and clinical signs of SFN, aged 18–75 years, were included. The guideline of the American Thoracic Society was used for the diagnosis of sarcoidosis.^20^ Exclusion criteria were large fiber neuropathy, other diseases with a risk for developing (poly)neuropathy or SFN, diseases affecting sensory nerve function, pregnancy, psychological problems, language barrier, glucose intolerance, rheumatoid arthritis, vitamin B12 deficiency, glaucoma, cataract, contact lens wear, anterior chamber angle of the eye below grade 2, keratoconus and excessive alcohol consumption, as assessed by the treating physician.

### Ophthalmologic assessment

An ophthalmologist examined the participants to assess eye involvement in sarcoidosis and general eye condition for exclusion criteria. Glaucoma, keratoconus, and uveitis were assessed by measuring visual acuity, best visual acuity, slit lamp examination, and fundoscopy. The anterior chamber angle of the eyes was determined using the Van Herrick technique.

### Neuropathy assessment

Neuropathy assessment was performed as previously described.^21^ Briefly these include a diagnosis of ‘probable SFN’ based on symptoms, clinical signs during neurological evaluation (presence of 2 or more clinical signs such as hypoalgesia, allodynia, hyperalgesia, and thermal hypesthesia, see Table 1 for all symptoms), and normal nerve conduction studies, as defined according to the Besta criteria.^12^

**Table 1:** Symptoms related to small fiber neuropathy

General Symptoms	Sensory disturbances	Autonomic dysfunction
Fatigue	Neuropathic pain	Skin changes
Cognitive disturbances	Burning sensations	Sweating abnormalities
Headache	Paresthesia	Vision
Widespread musculoskeletal pain	Hypesthesia/numbness	Urinary tract
	Tingling	Dry mouth
	Itching	Gastrointestinal dysmobility
	Frostbite-like sensations	Orthostasis
	Bedsheet/clothing intolerance	Palpitations
	Stocking-glove/random/migratory and/or intermittent	Bowel or bladder changes
	Hot flushes	Sexual dysfunction

### SFN phenotyping questionnaire

By introducing a new SFN phenotyping questionnaire (SFNPQ), we distinguished patient-reported length-dependent, non-length-dependent, intermittent, and continuous pain. In addition, we identified the anatomical level of pain, see Appendix 1 for the full questionnaire.

**Figure 1:**
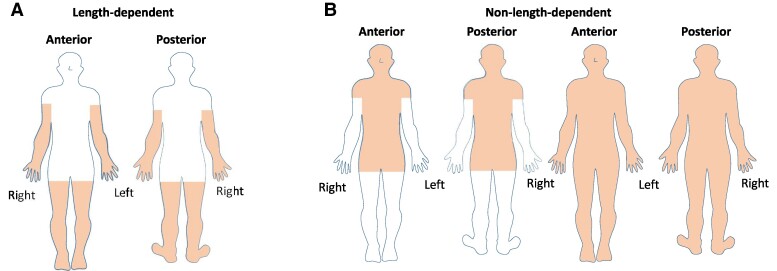
**Pain phenotypes.** Definition of (**A**) length-dependent and (**B**) non-length-dependent pain phenotypes.

The newly developed SFNPQ consisted of three different questions:

1. Do you feel pain intermittently or continuously?
  - intermittently
  - continuously
2. Please identify the anatomical level of pain:
  1. pain in skin
  2. pain in muscles
  3. pain in joints
  4. fill in: pain in …
3. Please mark the anatomical location of pain in the figure below, see Fig. 1. Mark only the areas that apply to you.

Participants were required to use a blue pencil for intermittent pain and a red pencil to locate continuous pain. Intermittent pain was defined as any pain that is not continuously present on a daily basis. Figure 1 is displayed four times, each representing one anatomical level as mentioned in the second question. This questionnaire was completed independently by the patients. In addition to the SFNPQ, the SFNSL was administered.^19^

### Phenotypes of patient-reported pain

Length-dependent and non-length-dependent phenotypes were distinguished based on patient-reported pain symptoms. A length-dependent phenotype was defined as pain starting in the feet and legs with a possible combination of pain in the hands and arms (Fig. 1A). A non-length-dependent phenotype could manifest as pain anywhere except the extremities, or in combination with symptoms in the extremities (Fig. 1B). Only pain at the skin level could be associated with length-dependent neuropathic pain. This study therefore only shows the results from the first figure indicating neuropathic pain, see Supplementary material for the questionnaire. Results from the questionnaire at muscle, joint, and other levels (Supplementary Figs 2–4) were beyond the scope of this article and therefore not shown in the results section.

### Data analysis

First, the prevalence of patient-reported length-dependent and non-length-dependent pain was determined without distinction between intermittent and continuous pain. Next, the prevalence of patient-reported intermittent and continuous pain was determined by categorizing four phenotypes:

1. intermittent length-dependent pain;
2. intermittent non-length-dependent pain;
3. continuous length-dependent pain; and
4. continuous non-length-dependent pain.

The presence of patient-reported length-dependent and non-length-dependent presentation was compared between intermittent and continuous symptoms. Statistical significance was calculated using odds ratios (OR) and chi-square tests. Patients could have both intermittent and continuous pain at the same time, but for one type of pain (intermittent or continuous), the presentation could be length-dependent or non-length-dependent.

Finally, the association between diagnostic methods and patient-reported pain was examined. Therefore, the correlation between the four phenotypes, the SFNSL questionnaire, TTT, IENFD, CCM, Sudoscan, and water immersion skin wrinkling (WISW) was assessed.

### Quantitative sensory testing

The Medoc Thermal Sensory Analyser 2 (TSA2) was used to assess sensory nerve function. TTT was performed on both feet to assess small nerve fibers. The German Research Network on Neuropathic Pain developed a standard operating file^22^ that was used to instruct participants. Moreover, their cutoff values were used to define abnormal parameters.^23^ The number of abnormal TTT parameters (TTT NOAs) was used as a parameter for further analysis.^21^

### Skin biopsy

Three mm punch biopsies were collected 10 cm above the lateral malleolus of the right leg to determine IENFD. For automatic PGP9.5 staining, 10 µm sections were used as previously described.^24^ According to the guidelines of the European Federation of Neurological Society (EFNS),^25^ this method shows comparable results to the recommended method based on 50 µm sections. Four consecutive sections separated by 100 µm were analyzed with a light microscope at 400 × magnification.

Because high-resolution scans are currently available, we counted all nerve fibers in the entire sample. The IntelliSite Image Management System (IMS v.1.8.6824, Philips Healthcare, Amsterdam, the Netherlands) was used to calculate the entire surface of the epidermis. We counted all nerve fiber fractions with a minimal length of 5 µm.^26^ When fractions were aligned on the same trajectory, they were counted as one nerve, as long as the distance between fractions did not exceed the length of the longest nerve fraction in that trajectory.

### Corneal confocal microscopy

The Heidelberg Retina Tomograph III with Cornea Rostock Module (Heidelberg, Germany) was used to image corneal nerve fibers. This allowed a corneal surface of 400 × 400 µm to be photographed with a resolution of 384 × 384 pixels. Corneal imaging was performed according to a publicly available protocol.^27^ CNFD, cornea nerve branch density (CNBD), and cornea nerve fiber length (CNFL) were calculated using automatic software (ACCMetrics).^28^ Images from both eyes were selected by visual inspection of their quality. Age- and sex-stratified reference values from healthy volunteers were used to define impaired corneal parameters.

### Sudoscan

Electrochemical skin conductance (ESC) was measured on both hands and feet with the Sudoscan® (Impeto Medical, Paris, France) to assess autonomic sudomotor function. Two stainless steel electrodes generated a low-voltage electric current. ESC (µS) for palms and soles was automatically calculated by the Sudoscan computer. Lower limits of 57 µS for the hands and 71 µS for the feet were used.^29^

### Water immersion skin wrinkling

Water immersion skin wrinkling was used to assess another aspect of autonomic function. The right hand was placed in water at 40 ֯C for 30 min. Photos were taken afterward to store in a database. The degree of wrinkling on the right hand was compared with the left hand as a reference. Wrinkling was graded between no wrinkling (grade 0) and wrinkling with complete deformation of the fingertip (grade 4).^30^

### Statistics

SPSS (v26, IBM, Armonk, NY, USA), Rstudio (v4.3.1, Rstudio, Boston, MA, USA), and Graphpad Prism (v8.4.3, GraphPad Software, San Diego, CA, USA) were used for statistical analysis. The odds ratio was used to determine the statistical significance between the prevalence of intermittent and continuous pain with a length-dependent and non-length-dependent presentation. Spearman’s rank correlation coefficient was used to assess the association between phenotypes and diagnostic parameters. Finally, the Mann–Whitney U-test was used to determine statistical significance between TTT NOAs in patients with and without continuous length-dependent pain.

## Results

### Patient selection

**Figure 2:**
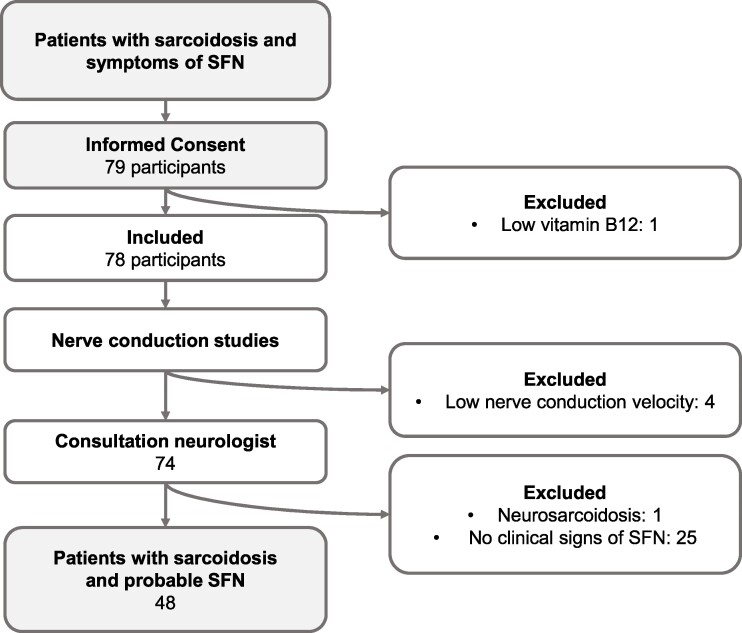
**Inclusion process.** Schematic overview of inclusion and clinical diagnosis of SFN in this study. A total of 79 participants provided informed consent, of whom six were excluded. One participant showed low vitamin B12 levels, four patients were excluded based on low nerve conduction velocity, and one was diagnosed with neurosarcoidosis. Of the 74 patients with sarcoidosis and symptoms of SFN, 48 were diagnosed with probable SFN. Abbreviations: SFN, small fiber neuropathy.

A total of 79 patients with sarcoidosis participated in this study, of whom 48 could be diagnosed with probable SFN (Fig. 2). Demographic data are shown in Table 2.

**Table 2:** Patient characteristics in healthy controls and patients with sarcoidosis

Group	Sarcoidosis with SFN
*n*	48
Age (mean yrs ± sd)	52 ± 9
Sex Males (*n* (%))	21 (44%)
Height (mean ± sd)	176 ± 11
BMI (mean ± sd)	28 ± 6
Disease duration sarcoidosis (mean yrs ± sd)	9 ± 7

BMI, body mass index; SFN, small fiber neuropathy.

### Symptoms mentioned during neurological consultation

An overview of SFN-related symptoms reported by the participants is shown in Table 3. Pain, burning sensations, and paresthesia were most frequently prevalent and observed in 50%, 44%, and 42%, respectively.

**Table 3:** Overview of small fiber neuropathy (SFN) symptoms in patients with sarcoidosis. Absolute numbers of symptoms and percentages were displayed

SFN symptoms	Present (*n*(%))
Pain	24 (50%)
Burning sensations	21 (44%)
Paresthesia	20 (42%)
Bedsheet/clothing intolerance	10 (21%)
Headache/dizziness	8 (17%)
Hypesthesia/numbness	6 (13%)
Gastrointestinal	6 (13%)
Urinary tract	5 (10%)
Cold sensation	4 (8%)
Hot flushes	4 (8%)
Vision	3 (6%)
Orthostatic hypotension	2 (4%)

### Phenotypes of SFN

**Figure 3:**
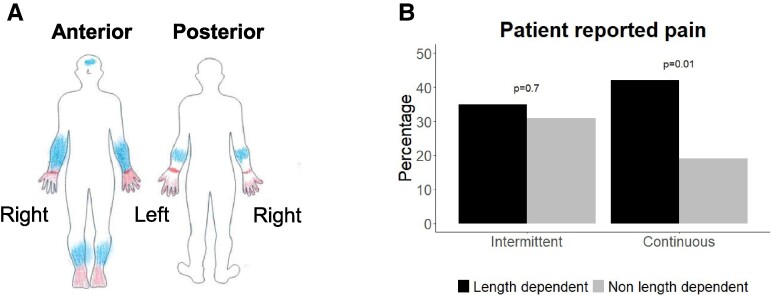
**Results of the phenotyping questionnaire.** (**A**) Example of a completed small fiber neuropathy phenotyping questionnaire (SFNPQ). It shows length-dependent continuous pain in combination with non-length-dependent intermittent pain. (**B**) Prevalence of length-dependent and non-length-dependent pain for continuous and intermittent presentations (*n* = 48). Continuous non-length-dependent pain was significantly less reported, (OR = 0.3, *P* = 0.01, chi-square tested).

Figure 3A shows an example of one participant’s completed SFNPQ. Of the 48 patients with probable SSFN, 17 (35%) showed a length-dependent and 21 (44%) a non-length-dependent phenotype (OR = 1.4, *P* = 0.4). Of the 21 patients with a non-length-dependent pain presentation, six reported no pain in the limbs at all. The 10 (21%) patients without cutaneous pain all suffered from muscular or articular pain. For these results, no distinction was made between intermittent and continuous pain. Therefore, the example presented in Fig. 3A was identified with a non-length-dependent phenotype.

Subsequently, a distinction was made between intermittent and continuous pain. Out of 48 patients with probable SSFN, 13 (27%) patients reported intermittent pain, 11 (23%) continuous pain, and 14 (29%) mixed pain. According to these criteria, the example presented in Fig. 3A was classified as intermittent non-length-dependent pain in combination with continuous length-dependent pain.

Figure 3B shows which of the four phenotypes were more commonly reported by patients with probable SSFN. Intermittent pain was reported with both, length-dependent and non-length-dependent presentation without significant difference between the two groups (OR = 0.8, *P* = 0.7), while continuous pain was reported more often as length-dependent presentation (OR = 0.3, *P* = 0.01, Fig. 3B).

**Figure 4:**
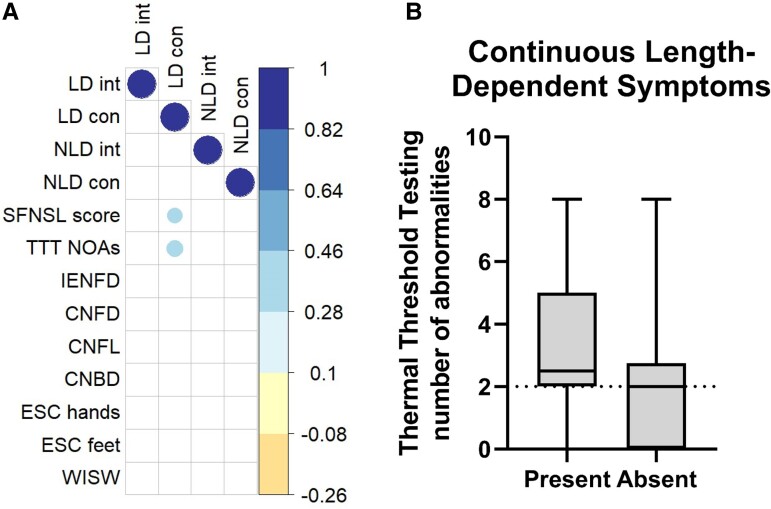
**Correlation plot.** (**A**) Correlation between patient-reported outcome measures and diagnostic methods for SFN. The correlation coefficient ranges between −1 for a negative correlation, 0 for no correlation, and +1 for perfect positive correlation (*n* = 48, ). (**B**) Boxplots with median TTT NOAs and min-max whiskers for patients with sarcoidosis and probable SFN, with (Present) and without (Absent) continuous length-dependent pain (*n* = 48, *P* = 0.02, Mann-Whitney U tested). Abbreviations: LD int, intermittent length-dependent pain; LD con, continuous length-dependent pain; NLD int, intermittent non-length-dependent pain; NLD con, continuous non-length-dependent pain; SFNSL, small fiber neuropathy screening list; TTT NOAs, thermal threshold testing number of abnormalities; IENFD, intraepidermal nerve fiber density; CNFD, corneal nerve fiber density; CNFL, corneal nerve fiber length; CNBD, corneal nerve branch density; ESC, electrochemical skin conductance; WISW, water immersion skin wrinkling.

*Correlation between diagnostic methods and patient-reported phenotypes* Continuous length-dependent pain was the only patient-reported phenotype that correlated with TTT NOAs (*R* = 0.3, *P* = 0.02) and the SFNSL (*R* = 0.3, *P* = 0.05). Furthermore, TTT NOAs correlated with the SFNSL (*R* = 0.3, *P* = 0.03) (Fig. 4A). After dividing the participants into groups with and without continuous length-dependent pain, significantly higher TTT NOAs were found in the group with continuous length-dependent pain (Fig. 4B).

## Discussion

This study describes patient-reported outcomes in SFN-associated pain in a cohort of patients with sarcoidosis and probable SFN. SFN-associated pain in our cohort of patients with sarcoidosis was reported both to be length-dependent and non-length-dependent phenotypes. The most important and novel finding of our study is that TTT in patients with SSFN was only associated with continuous length-dependent pain.

Sarcoidosis is an immune-mediated inflammatory disease with an unknown cause. SFN-related pain in our cohort of sarcoidosis patients was reported as both length-dependent and non-length-dependent. This contradicts previous results that claim more often non-length-dependent symptoms.^10,31^ However, SFN-related symptoms are more often non-length-dependent and patchy in patients with immune-mediated disorders, than in patients with metabolic diseases such as diabetes mellitus, who manifest mainly with length-dependent symptoms.^9,10^ A possible explanation is that glucose dysmetabolism probably affects distal nerve axons in the form of a ‘dying-back’ process and that immune-mediated diseases mainly affect sensory neurons in a more random process.^10^ Furthermore, there may be a link between immune-mediated diseases and inflammatory dorsal root ganglionitis, which affects the small sensory neurons of the dorsal root ganglia.^32^

Commonly used and well-established diagnostic criteria for SFN include IENFD and TTT.^25^ Because both methods are performed on distal parts of the extremities, it remains to be seen whether these tests are most suitable for SSFN, which based on our results is non-length-dependent in more than 40% of patients with sarcoidosis. CCM can be used to determine CNFD of the sensory terminals of the trigeminal nerve in the cornea. Because the trigeminal nerve is a short nerve, it has been suggested that decreased CNFD could be informative in patients with non-length-dependent SFN. We found no difference between CNFD in SSFN patients with a length-dependent or non-length-dependent phenotype. Other diagnostic tests, such as Sudoscan and WISW, also failed to distinguish between length-dependent and non-length-dependent phenotypes.

Because patient-reported pain in probable SSFN has never been classified as continuous or intermittent, we addressed this issue. Interestingly, we found a difference in TTT results when we assessed phenotypes based on length-dependent, non-length-dependent, continuous and intermittent pain. Patients with continuous length-dependent pain showed significantly more TTT NOAs than patients without continuous length-dependent pain. This indicates that TTT is likely prone to give false-negative results in patients with intermittent length-dependent or non-length-dependent pain. It is important to note that TTT alone is not solely sensitive for SFN. For example, abnormal TTT measurements can be found in patients with central nervous system disorders^33^ and are dependent on the patient's cooperation.^34^ To minimize these influences, we used strict exclusion criteria to ensure a normal central nervous system and normal large nerve fibers. As applicable to other neurophysiologic tests, TTT should always be interpreted in light of the patient’s clinical presentation. Therefore, identifying phenotypes of SFN could help interpret the combination of clinical presentation and TTT results.

Although the diagnostic criteria suggest that definite SFN can be diagnosed with abnormal TTT and/or decreased IENFD, decreased IENFD has been suggested as surrogate gold standard, based on the fact that it is an objective measurement.^35^ In clinical practice, the majority of SFN diagnosis relies on an abnormal TTT rather than decreased IENFD, revealing the low sensitivity of the latter test.^15,36-40^ For example, a recent large study described the role of both IENFD and TTT in the diagnostic trajectory of SFN in 243 patients.^40^ In this cohort, 50% of patients had decreased IENFD, while 90% of patients with SFN showed an abnormal TTT. Given that TTT plays an important role in the diagnosis of SFN in current clinical practice, regardless of the underlying cause, our findings regarding a higher diagnostic yield of TTT in patients reporting continuous length-dependent pain need to be further elucidated. If this is confirmed in other studies, it could be debated whether in-depth phenotyping of pain symptoms should be taken into account when using a diagnostic test such as TTT in the diagnostic trajectory of SFN, especially in patients with inflammatory disease who are likely to have a non-length-dependent phenotype.

In addition to distinguishing between length-dependent and non-length-dependent phenotypes, the SFNPQ skin pain data distinguished between continuous and intermittent pain. Of the four different phenotypes, continuous non-length-dependent pain was the only phenotype less reported in patients with probable SSFN. The fact that only continuous length-dependent pain correlates with TTT NOAs and the SFNSL implies that intermittent pain cannot be measured by any diagnostic method. It is important to note that only subjective measures correlated (continuous length-dependent pain, the SFNSL, and TTT NOAs), while none of the objective diagnostic methods did. Moreover, intermittent pain likely has different pathological mechanisms than continuous pain, which are relatively unknown and more difficult to understand and measure.

The results show that the diagnostic criteria, based on symptoms, clinical signs (dominant peripherally determined), and abnormal quantitative sensory testing at the feet and/or reduced IENFD, tend to be more sensitive for patients who report continuous length-dependent pain. The SFNSL questionnaire, although no diagnostic test, was validated against the diagnostic criteria and inherits the same bias.^19^ Therefore, awareness of this bias should be increased when validating new methods against these diagnostic criteria.

A limitation of our study was the lack of 50 µm IENFD for the assessment of SFN according to the EFNS guidelines^25^ and a limited sample size of patients with SFN. The thin tissue sections result in more fragmented nerve structures rather than entire nerve branches. Furthermore, the counting method was modified to count nerve fragments instead of whole structures. This might have contributed to the fact that no association was found between IENFD and a length-dependent phenotype in our cohort.

Another limitation is that only CCM was hypothesized to correlate with patient-reported non-length-dependent pain. For example, a proximal lower extremity biopsy could have been used to diagnose non-length-dependent SFN. Consequently, an association between patient-reported non-length-dependent pain assessed by skin biopsy and the SFNPQ cannot be evaluated. The proximal biopsy was not performed due to the unknown added value of the new staining protocol based on 10-µm sections.

In addition to proximal skin biopsy, the quantitative sensory axon reflex test (QSART) could have been used to assess non-length-dependent SFN. Like the Sudoscan, QSART assesses the sudomotor function. However, Sudoscan is only performed on the hands and feet, while QSART measures an indirect reflex-mediated sweat response over time at both proximal and distal sites of the limbs.^41^ Therefore, QSART would have been a useful addition to this study.^42^

A limitation of the SFNPQ is that it remains unclear whether the symptoms are actually related to SFN or not. Like the general symptoms presented in Table 1, the symptoms are not specific to SFN and may be present without any association with SFN. Therefore, we identified probable SFN based on the globally recognized Besta criteria in a well-defined cohort of patients with sarcoidosis while applying strict exclusion criteria.^12^ Despite this general limitation, the correlation between patient-reported continuous length-dependent pain and TTT NOAs remains particularly interesting.

The strength of this study is the use of diagnostic methods based on structural, functional, and autonomic function assessment for comparison with a new questionnaire using patient-reported outcomes. Multimodal testing for diagnosing SFN is known to improve diagnostic accuracy, but shows poor correlation between testing modalities.^15^ Phenotyping based on patient-reported outcome measures now provides a better understanding of what mechanisms can be measured using different diagnostic techniques.

The added value of the newly developed questionnaire is twofold. For research purposes, the SFNPQ identifies detailed patient-reported pain phenotypes that provide new insights into SFN-associated pain for specific underlying mechanisms. Patient-reported continuous non-length-dependent pain was less common in SSFN. Furthermore, the diagnostic criteria were less applicable in patients without continuous length-dependent pain. Further research is needed to identify the relationship between patient-reported pain and SFN and to investigate the pathophysiological mechanisms of intermittent pain. Determining the pathophysiological mechanisms could help develop new diagnostic methods.

## Conclusion

Patients with SSFN report both length-dependent and non-length-dependent pain and demonstrate a balanced presentation of intermittent and continuous pain. A novel finding is that TTT is only associated with continuous length-dependent pain, suggesting that TTT could result in more false negatives in patients with pain phenotypes other than continuous length-dependent. If this is confirmed in other cohorts, it will have implications not only for the diagnosis of SSFN but for all underlying diseases in which SFN-related pain presents non-length-dependent or intermittently.

## Supplementary Material

fcae289_Supplementary_Data

## Data Availability

Data access is restricted and can be made accessible through the corresponding author, upon reasonable request. Anonymized data supporting the findings of this study is available via this link 10.5281/zenodo.11047637.
