# Supplementary material for: New phenotyping questionnaire for diagnosing sarcoidosis-associated small fiber neuropathy
Source: Brain Commun. 2024 Aug 28;6(5):fcae289. doi: 10.1093/braincomms/fcae289 (PMC11406462; doi:10.1093/braincomms/fcae289)

Supplementary material

Small fiber neuropathy phenotyping questionnaire

This questionnaire is an addition to the small fiber neuropathy screening list and focusses on pain symptoms.

In which organs do/did you have sarcoidosis granulomas?:

- ☐ Lungs
- ☐ Heart
- ☐ Nerves
- ☐ Other:………………………………….

Can you indicate whether you experience your pain continuous, intermittent or both?

- ☐ Continuous
- ☐ Intermittend, for example provoked by warm socks or physical exertion

In the following figures we ask you to indicate where in the body pain occurs. If the pain is continuous, color that part with red and if the pain occurs in attacks, color that part blue/black. Since the body is made up of several organs, we ask you to distinguish between:

1. Pain in skin
2. Pain in muscles
3. Pain in joints
4. Fill in….

You can complete point 4 yourself if the pain occurs in a different location than mentioned under 1 to 3. You only need to color the figures that apply to you.

1. **Pain in skin**

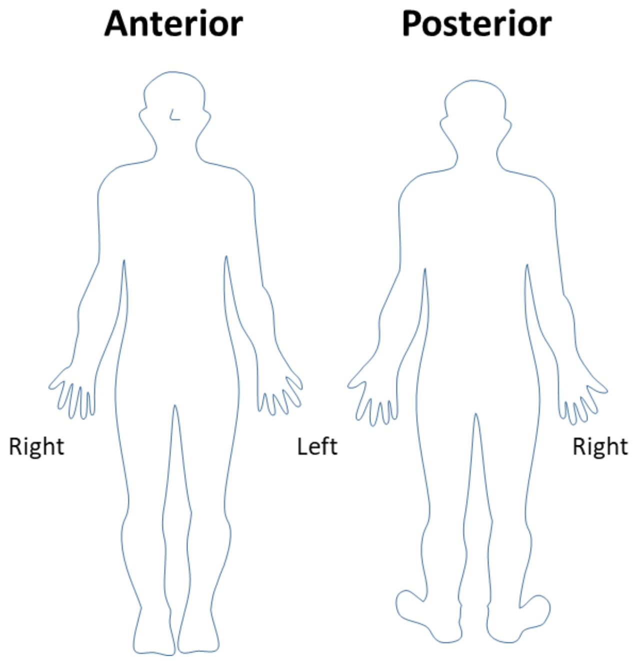

2. **Pain in muscles**

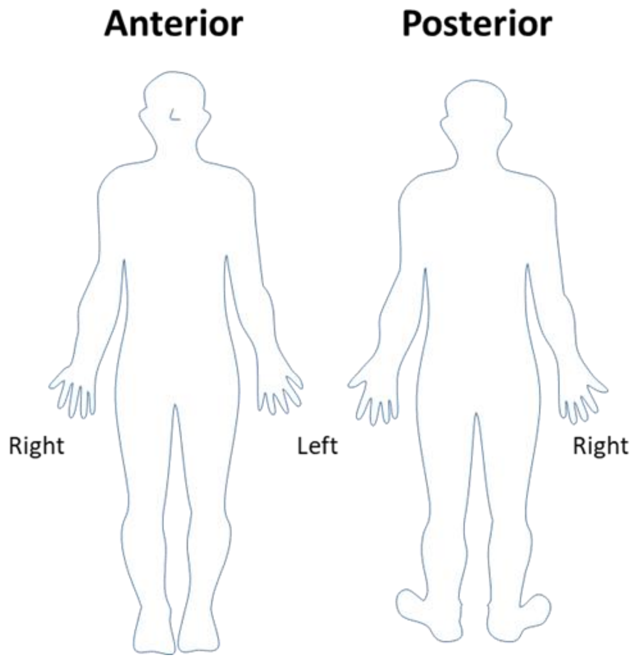

3. Pain in joints

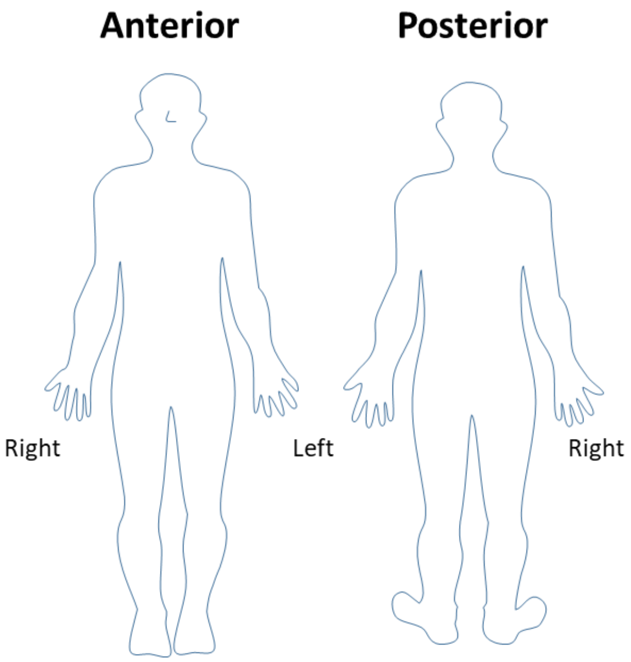

4. Pain in ……………………..

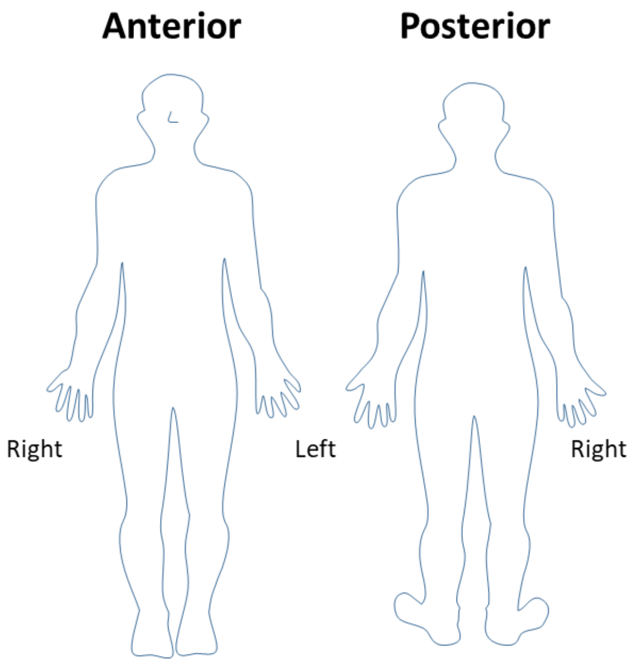

Supplement: fcae289_Supplementary_Data [file fcae289_supplementary_data.pdf]
